# Novel Therapeutics in Radioactive Iodine-Resistant Thyroid Cancer

**DOI:** 10.3389/fendo.2021.720723

**Published:** 2021-07-15

**Authors:** Tanner Fullmer, Maria E. Cabanillas, Mark Zafereo

**Affiliations:** ^1^ Department of Head and Neck Surgery, The University of Texas MD Anderson Cancer Center, Houston, TX, United States; ^2^ Department of Endocrine Neoplasia and Hormonal Disorders, The University of Texas MD Anderson Cancer Center, Houston, TX, United States

**Keywords:** thyroid cancer, iodine resistance, radioactive iodine resistance, novel therapeutic, well differentiated thyroid cancer, anaplastic thyroid cancer

## Abstract

Iodine-resistant cancers account for the vast majority of thyroid related mortality and, until recently, there were limited therapeutic options. However, over the last decade our understanding of the molecular foundation of thyroid function and carcinogenesis has driven the development of many novel therapeutics. These include FDA approved tyrosine kinase inhibitors and small molecular inhibitors of VEGFR, BRAF, MEK, NTRK and RET, which collectively have significantly changed the prognostic outlook for this patient population. Some therapeutics can re-sensitize de-differentiated cancers to iodine, allowing for radioactive iodine treatment and improved disease control. Remarkably, there is now an FDA approved treatment for BRAF-mutated patients with anaplastic thyroid cancer, previously considered invariably and rapidly fatal. The treatment landscape for iodine-resistant thyroid cancer is changing rapidly with many new targets, therapeutics, clinical trials, and approved treatments. We provide an up-to-date review of novel therapeutic options in the treatment of iodine-resistant thyroid cancer.

## Introduction

Thyroid cancer is the most common endocrine malignancy and its incidence has been increasing over the last several decades. Differentiated thyroid cancer (DTC) makes up approximately 90% of all thyroid cancer and overall has excellent 10-year disease specific survival approximating 95% (1). These include papillary thyroid cancer (PTC), follicular thyroid cancer (FTC), and Hurthle cell variants. Most of these tumors are isolated to the thyroid gland, though regional disease at presentation is quite common for papillary and Hurthle cell thyroid carcinomas. These patients do well with thyroidectomy and central or lateral compartment neck dissection as needed for radiographically evident disease. Radioactive iodine (RAI) is administered in those with intermediate to high risk features and other patient factors (2).

However, approximately 10% of those with DTC will present with distant metastasis and an additional 6-20% will recur at distant sites (3–6). The standard of care for these patients is surgery to control recurrent local/regional disease, followed by evaluation for RAI. The overall 10-year survival for those presenting with distant metastasis is much less at approximately 50% (6). Additionally, about 2/3^rd^s of patients with DTC and distant metastasis may show de-differentiation and decreased iodine uptake, making adjuvant treatment with radioactive iodine ineffective. Durante et al. has shown the 10-year overall survival for this cohort is approximately 10-20% (7) while those who respond to RAI may still have 10 year survival approaching 90% (3, 7). Metastasis to sites other than the lung also portends a poorer prognosis as these have even higher rate of resistance to iodine. Unfortunately, these tumors are also frequently insensitive to traditional cytotoxic chemotherapy agents.

Poorly differentiated thyroid cancer (PDTC) and anaplastic thyroid cancer (ATC) are a subset of thyroid carcinomas that also derive from thyroid follicular cells i.e. the cells responsible for iodine concentration and thyroid hormone production. They lie on the spectrum of progressive loss of typical features and function of normal thyrocytes. Many of these tumors have no ability to concentrate iodine and cannot be treated with RAI (8). Anaplastic thyroid cancer (ATC) is the most aggressive form of thyroid cancer with a historic median survival of approximately 6 months (9). Fortunately, ATCs are rare, accounting for approximately 2-5% of thyroid cancers. Recently, the prognosis patients with ATC has improved considerably (9). Medullary thyroid carcinoma is derived from the parafollicular C cells and is foundationally different than iodine resistant DTC, PDTC, and ATC, and consequently its treatment is not detailed specifically here.

Radioactive iodine-resistant/refractory (RAI-R) thyroid cancers (TC) account for the majority of thyroid cancer associated mortality. Fortunately, the molecular underpinnings of the thyroid’s synthetic function and iodine metabolism as well as the identification of several important pathways involved in tumorigenesis has led to multiple therapeutic targets and revolutionized the treatment of this difficult disease. This has drastically changed the treatment and outlook of RAI-R TC in the last 10 years. In fact, the first therapeutic in the treatment of iodine resistant thyroid cancer was FDA approved in 2013. The landscape of FDA approved therapeutics, clinical trials, and potential areas of research is changing rapidly. This review will summarize the most current novel therapeutics available.

## Molecular Mechanism

Iodide is a fundamental component of thyroid hormone and is actively imported into thyroid follicular cells by a basilar membrane-bound protein, the sodium-iodide symporter (NIS) (10). A functional NIS is required for the active concentration of radioactive iodine within the thyroid gland where it is incorporated into colloid and degrades into beta and gamma rays causing cytotoxic DNA damage, apoptosis and cell death. Thus, the lack of expression or dysfunction of NIS has been hypothesized as an important contributor in iodine resistant tumors. Thyroid stimulating hormone (TSH) regulates the expression of NIS by stimulating the TSH receptor activating adenylyl cyclase and increasing the production of cAMP (10). This induces the transcription of NIS through thyroid specific transcription factors (TTFs) including paired box 8 (PAX8).

**Figure 1:**
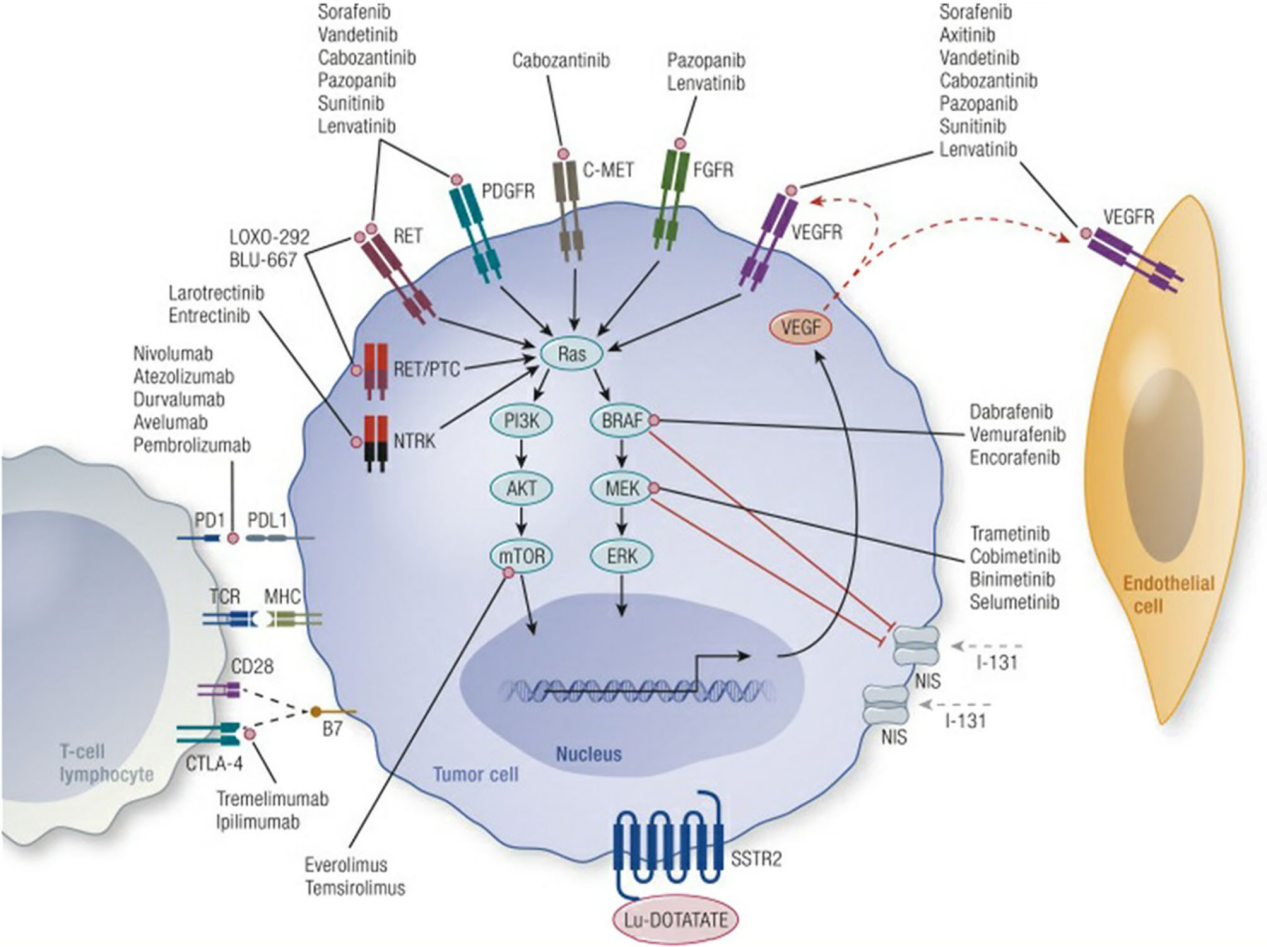
Mechanisms of tumor genesis in thyroid cancer and site of action of novel therapeutic agents. Credit Cabanillas et al., Targeted Therapy for Advanced Thyroid Cancer: Kinase Inhibitors and Beyond, Endocrine Reviews, 2019, 40:6, by permission of Endocrine Society, License# 4956001448885.

Genetic alterations within the mitogen-activated protein kinase (MAPK), and phosphoinositide 3-kinase (PI3K) pathways drive the pathogenesis of many differentiated thyroid cancers (**Figure 1**) (11). These pathways are activated by the binding of growth factors to upstream receptor tyrosine kinases (RTKs) including the RET proto-oncogene, VEGFR, and FGFR, see **Figure 1** (11). The most common mutations within these pathways include BRAF, RAS, PI3K, PTEN, and RET. Fusions which activate the MAPK pathway include NTRK, RET and ALK fusions.

Studies have shown that gain of function mutations within the MAPK can decrease NIS expression. The BRAF V600E activating mutation is the most well studied of these and can decrease NIS expression through the inhibition of TTFs including PAX8 and alterations in the NIS promoter region directly affecting its transcription (12–14). MEK is even further downstream of BRAF and directly plays a role in the expression of NIS (15). The PI3K pathway has also been shown to have an inhibitory function on NIS transcription. For example, RAS mutations that selectively stimulate PI3K can decrease TSH-induced NIS expression (16). Activation of mTOR also decreases NIS expression in thyroid cells (17). Alterations in other related pathways have also been shown to decrease NIS expression including loss of function mutations in PAX8 NIS promoter region mutations. Re-sensitization to RAI by inhibiting the suppression of NIS expression as described above is an exciting area of interest in the clinical treatment of iodine-refractory thyroid cancer.

In general, the mutational burden with thyroid cancer has been shown to be lower than in many other cancers. Still the genetic diversity of thyroid cancer can lead to multiple and competing parallel processes providing mechanisms for tumor escape from a targeted therapeutic. This can lead to an initial or developed drug resistance. For example, in a preclinical study, BRAF mutated cell lines treated with BRAF inhibitors that had developed a resistance had increased ligand-dependent signaling *via* human epidermal growth factor 2/3 (HER 2/3). Furthermore, inhibition of HER2/3 restored the anti-tumorigenic of BRAF inhibition (18). Additionally, BRAF inhibition has been shown to induce RAS mutations in PTC *in-vitro* studies and has been hypothesized as a tumor escape mechanism (19). This has been demonstrated clinically in a recent case series in patients receiving BRAF inhibitors who showed acquired RAS mutations at progression of their disease (20).

## Diagnosis of RAI-R Thyroid Cancer

According to the 2015 American Thyroid Association guidelines, well differentiated thyroid cancer is considered refractory to iodine when the malignant or metastatic tissue does not ever concentrate RAI, when it loses the ability to concentrate RAI, when RAI is concentrated in some tissue and not others, or when metastatic disease progresses despite the ability to concentrate RAI (2). An exact definition remains controversial and multiple specific descriptions of RAI-R DTC have been proposed and are summarized in **Table 1**. Indeed, a recent editorial in 2019 suggested the lack of consensus definition of RAI-R DTC as a major area of controversy between the ATA, European Thyroid Association, The European Association of Nuclear Medicine, and the Society of Nuclear Medicine and Molecular Imaging. A consortium with representatives from each of the respected societies identified 5 common clinical scenarios that suggest RAI-R disease, but again no single criterion alone should be used taken in isolation to determine treatment decisions (21). Instead the amount of RAI uptake on post therapy whole body scans (WBS) can be weighed against the total dose of RAI a patient has received, how well they tolerated the side effects, and tumor response to previous RAI treatments among other factors. The Response Evaluation Criteria in Solid Tumor (RECIST) has been also used and suggested as an objective method to categorize patients’ response to various treatments including RAI. Though an exact definition is somewhat less critical, establishing a threshold when a novel therapeutic should be considered continues to be an important research objective and warrants further discussion.

**Table 1:** Iodine Refractory Differentiated Thyroid Cancer Definitions.

Source	Definition
*ATA*	Disease that never concentrates radioactive iodine. Disease that loses ability to concentrate radioactive iodine. Radioactive Iodine is concentrated in some lesions, but not others. Disease progression despite significant radioactive iodine uptake.
*Joint* *Statement*	No ^131^I uptake on diagnostic ^131^I scan. No ^131^I uptake on a ^131^I scan performed several days after ^131^I therapy. ^131^I uptake is only present in some but not other tumor foci. DTC metastasis progress despite ^131^I uptake. DTC metastasis despite a cumulative ^131^I activity of >22GBq (600mCi).
*SELECT* *Criteria*	One or more lesion that did not demonstrate any RAI uptake. Measurable lesions that had progressed within 12 months of RAI treatment per RECIST v1.1 despite showing RAI avidity at the time of pretreatment or posttreatment scan. Patients received a cumulative activity of RAI >600 mCi (22 GBq) with last treatment in the 6 months.
*DECISION Criteria*	At least one target lesion without RAI uptake. Disease with uptake that progressed after one RAI treatment within 16 months Disease that progressed after two RAI treatments within 16 months Patients received a cumulative activity of >600 mCi (22 GBq)

It is also important to identify patients that may have an increased likelihood of harboring iodine refractory disease, as these patients may be candidates for early intervention. Risk factors for RAI-R TC include increasing age, Hurthle cell histology, high metastatic burden, metastasis outside the lung, and increasing FDG avidity on positron emission tomography/computerized tomography (PET/CT) (2, 4, 22). BRAF mutations increase the likelihood of iodine resistance due to suppression of NIS (23, 24). A rapid thyroglobulin (Tg) doubling time after thyroidectomy or RAI, or a Tg that does not respond appropriately after RAI may indicate a greater possibility of refractory disease (25).

Molecular testing and mutational mapping have emerged as possible adjuncts to imaging and pathology in differentiating aggressive disease. For example, BRAF V600E and RAS mutations in combination with telomerase reverse transcriptase (TERT) mutations have been linked to aggressive disease and de-differentiation (26). Furthermore, PTC can be classified into a BRAF-like subtype, or RAS-like based on the predominant mutational profile. BRAF-like mutations typically portend a worse clinical course (27). Still the Cancer Genome Atlas found that 97% of PTC harbor mutations, and although research into the prognostic value of mutational mapping is robust, it currently cannot be used to predict DTC response to RAI with an acceptable degree of fidelity.

The indications and methodology for molecular testing to direct targeted therapy is an active area of research and a detailed description is beyond the scope of this review. Broadly speaking, patients with advanced or uncontrolled DTC and PDTC who will likely require systemic therapy in the near future warrants mutational mapping of their tumors. ATC of any size with or without distant metastasis should be investigated for common somatic mutations, particularly *BRAF* V600E (9), as soon as it is diagnosed, as it will determine eligibility for selective kinase inhibitors. The most recently obtained specimen should be tested.

The easiest and least expensive method to identify *BRAF* V600E mutated tumors is immunohistochemistry (IHC), which can quickly be utilized on FNA cell blocks or surgical specimens (27–29). IHC has also been described for other mutations including RAS, ALK, RET though these are not currently utilized in practice. Single mutational testing may also be accomplished with any validated method including polymerase chain reaction, mass spectrometry, fluorescence *in situ* hybridization etc. Commercially available next generation-sequencing (NGS) assays are available for a more comprehensive tumor profile (27, 28). Mutational testing may be done in a multi-step iterative approach; however, comprehensive identification of multiple actionable rearrangements/mutations is common in practice as it requires less tissue and time.

## Treatment Decisions

A complete treatment decision hierarchy is beyond the scope and objective of this review, however, a brief description of appropriate management strategies of RAI-R TC is required to understand the indication for novel therapeutic use. As described, the majority of RAI-R TC occurs in the setting of advanced or recurrent disease, often with distant metastasis.

In potentially curable DTC with or without regional disease the preferred algorithm begins with partial or total thyroidectomy and, as indicated, a therapeutic central and/or lateral neck dissection. Nodal dissection is typically recommended for positive or suspicious nodes located on anatomic imaging (2). Following surgery, most patients will receive TSH suppressive doses of levothyroxine. In iodine-sensitive disease, this may be followed by RAI. Ethanol ablation may be considered in select cases of locoregional recurrence/persistent disease.

DTC/PDTC patients with residual or distant metastases are usually observed with TSH suppression alone to determine the pace of growth of their disease, as most patients will have indolent disease. Patients with progression within 6-12 months, or with disease that may cause morbidity if there is growth are considered for systemic therapy with kinase inhibitors. Clinically significant disease (≥1.5 cm) should also be present since very small disease is unlikely to cause morbidity (30). In patients with oligometastatic/oligoprogressive disease, localized therapy with Stereotactic body radiation therapy or gamma knife can be considered.

There have been major advances in the treatment of ATC over the last several years (31) as recent studies have demonstrated significant efficacy of BRAF/MEK inhibitors for BRAF-mutated ATC (9). Rapid and upfront genetic molecular testing, particularly for the BRAF V600E mutation, is mandatory. ATC patients with significant disease burden (vast majority of patients with ATC) and BRAF V600E mutation should be rapidly started on BRAF/MEK inhibitor therapy. Emerging data suggests that neoadjuvant targeted therapy with BRAF/MEK inhibitor may induce a significant and rapid therapeutic response allowing for definitive surgical resection in many cases, generally followed by resumption of BRAF/MEK inhibitor therapy (9, 32, 33).

Tumor Genetic molecular testing and potential systemic targeted therapy should be considered universally in patients with BRAF wild type (i.e. BRAF negative) ATC, with RET fusions, ALK fusions, and NTRK fusions representing other potential targetable mutations. Multikinase inhibitors (e.g. lenvatinib) and immunotherapy have also been variably associated with some treatment response in patients with ATC. For BRAF wild type ATC, upfront surgery is favored when possible, although often ATC patients present with locoregionally advanced disease which is not meaningfully surgically resectable (34). Significant burden of distant disease, particularly imminently threatening disease such as brain metastasis may dampen enthusiasm for surgical management of the neck and primary tumor. Radiation therapy with potential concurrent cytotoxic chemotherapy is another option for attempt at some degree of locoregional control in patients with unresectable BRAF-negative disease, or for whom surgery is not a meaningful option.

## Novel Therapeutics

Patients with symptomatic or progressive locoregional or metastatic RAI-R TC should be considered for targeted systemic agents. Over the last 10-15 years, targeted therapies have become a mainstay of treatment of RAI-R TC, namely tyrosine kinase inhibitors designed to target specific proteins within the MAPK, PI3K pathways among others and/or target upstream RTKs. Several of these drugs are specific to non-mutated proteins, while others target a particular alteration or mutation within these pathways. Thus, the latter may only be effective if the tumor contains the mutation, while the former may have a wider therapeutic indication. For structural consistency, the targeted therapies are organized into multi-target tyrosine kinase inhibitors and specific protein inhibitors (e.g., BRAF inhibitors). Multi-kinase inhibitors target multiple upstream RTKs and consequently have less specificity, potentially a broader application, and may have more off-target side effects. Specific protein inhibitors (BRAF, MEK etc.) are also kinase inhibitors, but they have fewer therapeutic targets and therefore more specificity. Currently available FDA-approved and non-FDA approved targeted therapeutics are summarized in **Table 2**.

**Table 2:** Novel Targeted Therapeutics in Iodine Resistant Thyroid Cancer.

Therapeutic	Target	Indication	Best evidence
**FDA Approved for Thyroid Cancer Treatment**			
*sorafenib*	VEGFR 1-3, PDGFR, RET, c-KIT, BRAF	Locally recurrent or metastatic, progressive, differentiated thyroid carcinoma refractory to RAI	Phase III (35)
*lenvatinib*	VEGFR 1-3, FGFR 1-4, PDGFR, RET, c-KIT	Locally recurrent or metastatic, progressive, differentiated thyroid carcinoma refractory to RAI	Phase III (36)
*dabrafenib and trametinib*	BRAF/MEK	BRAF V600E mutated anaplastic thyroid cancer with no satisfactory locoregional treatment options	Phase II (37)
*selpercatinib*	RET	RET-mutant medullary thyroid cancer, RET fusion-positive thyroid cancer refractory to RAI	Phase II (38)
*pralsetinib*	RET	RET-mutant medullary thyroid cancer, RET fusion-positive thyroid cancer refractory to RAI	Phase II NCT03037385
*larotrectinib*	TRKs	Metastatic or unresectable solid tumors with NTRK gene fusions and no alternative treatments	Phase II (39, 40)
*entrectinib*	TRKs, ALK, ROS	Metastatic or unresectable solid tumors with NTRK gene fusions and no alternative treatments	Phase II (41)
*cabozantinib*	VEGFR, MET, RET, AXL	Medullary thyroid cancer	Phase II/III
*vandetanib*	VEGFR, EGFR, c-KIT, RET	Medullary thyroid cancer	Phase II/III
**Other FDA Approved Therapeutics with Off-Label Evidence or Ongoing Trial in RAI-R TC**			
*sunitinib*	VEGFR 1-2, PDGFR, CKIT, RET, CSF1R	Advanced DTC resistant to RAI	Phase II (42)
*pazopanib*	VEGFR, FGFR, PDGFR, RET, c- KIT	Advanced DTC resistant to RAI, no effect on ATC	Phase II (43, 44)
*axitinib*	VEGFR, PDGFR, c-KIT	Advanced DTC resistant to RAI, limited ATC data	Phase II (45)
*anlotinib*	VEGFR, PDGFR, FGFR	Advanced DTC resistant to RAI, ATC	Preclinical Phase II pending (NCT02586337)
*cabozantinib*	VEGFR, MET, RET, AXL	Advanced DTC resistant to RAI who failed prior VEGF therapy	Phase II (46) Phase III pending (NCT03690388)
*vandetanib*	VEGFR, EGFR, c-KIT, RET	Advanced DTC resistant to RAI	Phase III (NCT01876784)
*donafenib*	VEGFR, PDGFR	Advanced DTC resistant to RAI	Phase II (47) Phase III pending (NCT03602495)
*dabrafenib*	BRAF	Advanced BRAF V600E mutated DTC resistant to RAI	Phase II (48)
*vemurafenib*	BRAF	Advanced BRAF V600E mutated DTC resistant to RAI, BRAF V600E mutated ATC	Phase II (49)
*encorafenib*	BRAF	Advanced BRAF V600E mutated DTC resistant to RAI	Preclinical Phase II pending (NCT04061980)
*selumetinib*	MEK	Increases iodine uptake in advanced mutated DTC resistant to RAI, no antitumor effects in clinical trial	Phase II/II (50–52), Phase II
*everolimus*	mTOR	Advanced DTC resistant to RAI, ATC	Phase II (53)
*lapatinib*	HER2	Advanced BRAF V600E mutated IRTC in combination with dabrafenib	Preclinical Phase I pending (NCT01947023)
*PRRT*	Somatostatin analog	Advanced DTC resistant to RAI	Phase II (54)
*pembrolizumab*	PDL-1	Advanced PDL-1 expressing DTC resistant to RAI, in ATC combined with chemotherapy with questionable results	Phase I (55, 56) Phase II
*nivolumab*	PD-1	Advanced DTC resistant to RAI/ATC, combined with ipilimumab	Phase II pending (NCT 03246958)
*ipilimumab*	CTLA4	Advanced DTC resistant to RAI/ATC, combined with nivolumab	Phase II pending (NCT 03246958)

### Multi-Target Tyrosine Kinase Inhibitors

Sorafenib is an orally available TKI that targets VEGFR 1-3, platelet derived growth factor (PDGFR), RET, KIT proto oncogene (c-KIT), BRAF among others. It was approved by the FDA in 2013 after the DECISION trial (35). In this phase III, multicenter, two-arm, double-blind trial, 417 patients with iodine resistant DTC were assigned to receive 400mg twice daily sorafenib (n=207), versus a placebo (n=210). Patients on sorafenib had significantly longer progression-free survival at 10.8 months compared to the placebo group at 5.8 months. Overall survival was not significant across groups, but patients assigned to the placebo arm could cross over to the treatment arm on progression. The objective response rate was 12.2% in the sorafenib group versus.5% in the placebo group, and all were partial responses. Additionally, those without an objective response had longer interval of stable disease in the sorafenib group. Adverse events included hand and foot rash (76.3%), diarrhea (68.6%), alopecia (67.1%) and rash (50.2%), fatigue (49.8%), hypertension (40.6%) among others. Serious adverse events occurred in 37.2% of patients receiving sorafenib versus 26.3% on placebo. These included secondary malignancy, pleural effusion, dyspnea. Dose interruptions occurred in 66.2% of those on sorafenib, while only 26.6% in the placebo group. Similarly the sorafenib cohort had more dose reductions (64.3% versus 33.8%) and withdrawals (18.8% versus 5.3%) compared to the placebo. One death from myocardial infarction was attributed to the study drug (35).

lenvatinib is another multi-target TKI that is approved by the FDA (2015) in the treatment of iodine refractory DTC. It is active against VEGFR1-3, FGFR1-4, PDGF, RET, c-KIT among other targets. The phase III SELECT trial was published in 2015 and randomized 261 patients to receive 24mg daily of lenvatinib and 131 patients to receive a placebo (36). The median progression-free survival was 18.3 months in those who received lenvatinib compared to 3.6 months in the placebo group. Nearly 65% of patients had a response to lenvatinib vs 1.5% in placebo group. Overall, 97.3% of patients experienced an adverse effect of any kind in the treatment arm, which included hypertension (67.8%), diarrhea (59.4%), fatigue (59%), weight loss, nausea among others. Nearly 60% of those in the placebo arm also had an adverse event. There were 6 deaths considered to be drug related. Eighty-two percent required a dose interruption due to side effects, 67.8% required a dose reduction, and 14% of patients had to discontinue the drug because of severe side effects (36). Most in clinical practice do not tolerate a 24mg dose, and patients are often started at 20mg or lower.

There are no randomized controlled trials directly comparing sorafenib with lenvatinib in the treatment of RAI-R TC, though multiple attempts have been made to compare the treatments indirectly based on the previous phase II-III data with mixed results (57, 58). Overall, their efficacy and side effect profiles appear comparable. Unfortunately, the response are not durable once patients stop taking the medications. Furthermore, resistance to TKIs has been reported after long term use, especially to sorafenib (59). Both lenvatinib and sorafenib have similar targeting profile, however, FGFR is only targeted by lenvatinib and some have hypothesized this as an important factor in preventing the development of long-term resistance to TKI therapies (60, 61). An update to the SELECT trial showed an average duration of response of at least 30 months in those that responded to lenvatinib and could tolerate continued use (61). Fortunately, most studies show that severe complications related to TKIs occur in the early course of treatment suggesting that responders who tolerate the medication in the short term will likely tolerate a more prolonged course (36, 61, 62). While both the SELECT AND DECISION trials report on BRAF and RAS mutation status, both drugs had a similar response rate regardless of mutation profile. Consequently, molecular testing is not necessary when prescribing lenvatinib or sorafenib for RAI-R DTC. This is primarily due to their targeting of multiple effector proteins upstream of BRAF, RAS etc. (see **Figure 1**), and hence their therapeutic profile can be applied more broadly.

Several other tyrosine kinases are FDA approved for the treatment of other cancers and have been shown in phase I or phase II clinical trials to be active against iodine-refractory DTC. These include sunitinib, pazopanib, axitinib, anlotinib, cabozantinib, donafenib, dovitinib. Others have recently reviewed the available phase I and phase II trial data extensively for these agents (4, 63, 64). The side effect profile seems to be similar across agents, but the cross-reactivity rate is not always predictable, so a patient may not tolerate one therapeutic, but can tolerate another. Hence a patient may be trialed on other medications within this same general class if tolerance to a certain medication is an issue. Among the most common adverse reactions includes hypertension, skin changes, diarrhea, and fatigue. TKI’s are considered chronic therapies, and thus it is important to effectively and proactively manage side effects (65). Cabanillas et al. recently published suggested treatment and de-escalation guidelines in the use of lenvatinib according to the adverse event and the CTCAE grade (common terminology criteria for adverse events) (65). Since the side effect profile is similar across other TKIs it may be reasonable to employ a similar strategy more broadly.

The importance of having multiple TKI agents is also evident when considering that many patients will develop a resistance to commonly utilized agents such as lenvatinib, sorafenib, and pazopanib after long term use (46, 66). There have been several suggested hypotheses regarding tumoral escape mechanisms, which usually involve the activation of alternate pathways including human epidermal receptor (HER), anaplastic lymphoma kinase (ALK) fusions, etc. (63, 67). In these situations, patients may be “salvaged” either with an additional TKI or other targeted therapy. Cabanillas et al. reported on a prospective series of 25 patients with iodine refractory DTC previously treated with one or more TKIs who failed these treatments for various reasons. They were then given cabozantinib and saw a 40% partial response rate, with an additional 52% of patients who had stable disease through the trial period (46). Dadu et al. showed retrospectively in 60 patients, that those who became resistant to first line sorafenib and then salvaged with an additional targeted therapy had significantly improved overall survival (58 versus 28 months) and progression free survival (11.4 versus 7.4 months) compared to those that continued on sorafenib or stopped treatment (66). Anlotinib is a new TKI with strong VEGFR/PDGFR/FGFR inhibition that has sparked considerable interest in thyroid cancer treatment among many other cancers. Several phase II/III trials examining its efficacy in RAI-R DTC are on-going (NCT02486350, NCT04309136), and a recent phase II trial in MTC showed durable antitumor activity (68).

Vandetanib and cabozantinib are two multi-kinase inhibitor which also target RET tyrosine kinase and both are FDA approved to treat metastatic medullary thyroid carcinoma. Cabozantinib has been shown effective in iodine refractory DTC that progressed after more traditional TKI therapy and preliminary vandetanib results show similar effects (46, 69). There are ongoing phase III clinical trials of vandetanib and cabozantinib as monotherapy in refractory DTC (NCT 01876784, NCT 03690388).

### BRAF V600E Inhibitors

The *BRAF* V600E activating mutation is arguably the most well understood and studied genetic alteration in thyroid cancer. It is most common in PTC with a reported incidence of approximately 30-80% (70–74). BRAF mutations area also frequently seen in PDTC and ATC (approximately 40%) (75). There are three BRAF inhibitors commercially available and FDA approved for the treatment of metastatic melanoma: dabrafenib, vemurafenib, and encorafenib. These therapeutics are specific to the *BRAF* V600E mutation and typically patients must harbor this mutation to receive benefit.

Vemurafenib as a single agent was evaluated in a phase II clinical trial in patients with progressive BRAF V600E PTC who had received (n=22) and not received TKIs (n=26). The TKI naive cohort had a 38.5% response rate with a 15.6-month progression free survival while the previously treated cohort had a 27.3% response rate with a PFS of 8.9 months (49). Grade 1 and 2 events were common including rashes, fatigue, alopecia, dysgeusia. Grade 3 or 4 adverse occurred in more than 60% of patients and included squamous cell skin cancer and lymphopenia (49).

Dabrafenib has also been studied in *BRAF* V600E mutated TC (48, 76). Falchook et al. treated 14 patients with BRAF mutated RAI-R TC with single agent dabrafenib. Four patients had a partial response, while 6 had stable disease and the mean progression-free survival was 11.3 months. The most common adverse events were skin papillomas and hyperkeratosis, but there were 2 treatment related serious adverse events including neutropenia and development of squamous cell carcinoma (76). Shah et al. recently presented the results of a phase II trial comparing dabrafenib (n=22) with combination dabrafenib/trametinib (n=24) in the treatment of BRAF mutated PTC. Response rates were similar between cohorts (approximately 50%) as was progression free survival (11 versus 15 months) (48).

Dabrafenib in combination with trametinib is FDA approved for *BRAF* V600E mutated ATC. In a phase 2 basket study, 16 patients with ATC were treated with the combination therapy of dabrafenib and trametinib. Sixty-nine percent of patients responded with a 12-month duration of response, progression free response and overall survival of 90%, 79%, and 80% (37). A subsequent update reported progression free survival and OS of 60 and 80 weeks (77). Toxicities were monitored in 100 patients as the majority included in the trial had pathologies other than ATC. Adverse events were minor but relatively frequent with the most common being fatigue (38%), pyrexia (37%), nausea at (35%) with most severe grade 3 and 4 adverse events were anemia (5%). Considering these remarkable results previously untreatable ATC, the FDA approved the combination therapy for treatment of BRAF V600E mutated anaplastic thyroid cancer.

Those with ATC and a significant response in the neck to dabrafenib and trametinib may be considered for surgery to remove the primary tumor and/or locoregional disease. Wang et al. reported on a series of 6 *BRAF* V600E mutated ATC patients who received neoadjuvant dabrafenib and trametinib followed by a R1 or R0 surgical resection (32). Analysis of the surgical specimen revealed 0-5% viability in most tumors, though one was still 50% viable. Each patient continued dabrafenib and trametinib after surgery. Remarkably 100% of patients were alive at 6 months, though 1 patient died at 8 months and 1 at 14 months from distant metastasis (32). The impact targeted therapy has had on treatment and prognosis of ATC has been dramatic. Maniakas examined 479 patients with ATC treated from 2000 to 2019 at MD Anderson Cancer Center and found the overall survival at 1 and 2 years has increased from 35% and 18% (2000 to 2013, n=227) to 59% and 42% (2017 to 2019, n=152) (9). Interestingly, within the cohort from 2017 to 2019 there were 20 patients treated with BRAF directed neoadjuvant targeted therapy followed by surgery. The 1-year overall survival was 94% in this group, an improvement of 45% over those who did not (p=.02). Additionally, the median overall survival was not reached within the time frame of the study.

Encorafenib is another FDA approved in the treatment of BRAF mutated melanomas and colorectal cancer, but no clinical data exists for its use in thyroid cancer. Currently a phase II clinical trial began in November 2020 to examine encorafenib combined with Binimetinib (MEK inhibitor) with or without the immunologic, nivolumab (NCT04061980).

### MEK Inhibitors

Mitogen-activated kinase (MEK)-1 and 2 acts downstream of BRAF and its selective inhibition has been demonstrated have antitumorigenic effects in cancers with an activated MAPK pathway. Trametinib has been approved by the FDA as a single agent in the treatment of V600 mutated melanoma and in combination with dabrafenib in the treatment of mutated metastatic melanoma and ATC. Trametinib in combination with the TKI pazopanib has recently been suggested in the treatment of DTC without a mutated BRAF-V600E, but results failed to show improvement over pazopanib alone (78). There is no clinical data supporting the use of trametinib alone in the treatment of IRTC, though some suggest it’s potential as a single agent modality (79). In DTC, the only MEK inhibitor that has been studied is selumetinib.

Selumetinib is FDA approved for neurofibromatosis type 1. Selumetinib was studied as a single agent in a phase II trial of 39 patients with iodine refractory thyroid cancer with and without follicular elements (50). There was only 1 partial response, while 54% maintained stable disease, and 28% had progressive disease. Progression-free survival was 33 weeks in those with BRAF V600E mutations and only 11 weeks in those without (50). Ho et al. demonstrated selumetinib, as a single agent, increased iodine uptake in a small cohort of iodine refractory thyroid cancer (51). However, Ho later went on to conduct a larger phase III-controlled trial of 233 DTC patients with high risk features randomized to either receive selumetinib or a placebo, and then both groups received RAI (52). In the initial analysis there were similar remission rates between groups (approximately 40%). At 18 months those who received selumetinib trended toward higher rates of complete remission, 46.7% versus 35.5% though this was not significant (p=.2131) (52).

### Selective RET Inhibitors

RET mutations occur in approximately 50% of MTC and RET fusions occur rarely in DTC (less than 10%), PDTC, and ATC (approximately 1%). Selpercatinib is a highly selective small molecule RET kinase inhibitor. In May 2020 selpercatinib was approved by the FDA for treatment of *RET*-mutated MTC, RAI-refractory *RET* fusion thyroid cancer and *RET* fusion non-small cell lung cancer. In a phase I-II trial patients with RET-mutated MTC (n=143) and previously treated non-medullary TC (n=19) were given selpercatinib (38). The TC cohort response rate was 79% and the 1-year progression free survival was 64%. It was generally well tolerated with the most common grade 1-2 reactions being dry mouth, hypertension and diarrhea. Only 2% of all the patients enrolled (n=531) had to discontinue treatment secondary to drug-related events (38). Pralsetinib is another selective RET inhibitor and was FDA approved on December 1^st^ 2020 for the treatment of RET mutated MTC and RET fusion-positive RAI-R TC who require systemic treatment. Its efficacy was demonstrated in ongoing clinical trial NCT03037385.

### mTOR Inhibitors

Most available targeted agents act on the MAPK pathway and the mechanisms of escape and resistance are not well understood. Several hypotheses revolve around the upregulation of upstream RTK and the activation of parallel or redundant pathways. Mammalian target of rapamycin (mTOR) is a distant downstream effector in the PI3K pathway and has several known and specific kinase inhibitors. Everolimus is an immunosuppressant and is FDA approved in the prevention of organ rejection and several solid organ cancers. In 2013 Lim et al. published a multicenter phase II trial of everolimus in 40 patients with metastatic thyroid carcinoma of all subtypes including PTC (40%), FTC (20%), MTC (22%), PDTC (3%), ATC (15%) (53). A confirmed response was only observed in 2 patients (including 1 ATC patient), though the disease control was excellent at 81% and the median PFS was 47 weeks. In general, it was well tolerated with mild to moderate side effects in most patients (53). This work was expanded by Hanna in 2018 with another phase II trial of similar design and results though this group also showed those with PI3K/mTOR/Akt mutations did better on everolimus (80). In a small subset ATC patients (n=6), 3 were positive for PI3K/mTOR/Akt mutations and had a PFS of 15.2 months compared to an overall PFS of 2.8 months for the entire ATC cohort (80).

### TRK Inhibitors

Tropomyosin receptor kinases (TRKs) are active in cell signaling in the MAPK pathway and are encoded by the neurotrophic receptor tyrosine kinase genes NTRK1-2 and usually only active in the central nervous system. However, chromosomal fusion events and rearrangements involving NTRKs have been implicated in multiple pediatric and adult cancers (81). These fusion products lead to chimeric TRK proteins and ligand independent downstream signaling and constitutively active function, driving tumor genesis.

Larotrectinib is a highly potent and selective inhibitor of all TRKs approved for the treatment of adult and pediatric patients with NTRK fusions. Drilon et al. recently enrolled 55 patients with various cancers and 17 unique NTRK fusion positive tumors into a phase I/II trial to receive larotrectinib (81). This included 5 patients with thyroid tumors. In total 75% of patients responded, including 13% with a complete response. Response rate was independent of fusion product. The treatment was generally well tolerated with Grade1-2 increases in liver transaminases, fatigue, vomiting, dizziness, and constipation being the most common complications. Later in the expanded phase I/II trial analysis to include an additional 104 TRK fusion cancer patients, (159 total patients) the objective response rate was 79% regardless of tumor type (39). In pooled data from these phase I/II trials, Cabanillas separately analyzed the 28 locally advanced or metastatic thyroid cancer patients with TRK fusions (40). The majority of patients had PTC (68%), followed by ATC (25%), and FTC (7%). The objective response rate for all DTC was 90%, and ATC was 29%. The duration of response at 12 months was 95% (40). Recently, Groussin et al. published a case report of a patient with metastatic RAI-R PTC that was found to have a TRK fusion gene. Treatment with larotrectinib was initiated and after 3 weeks iodine uptake was restored. However, RAI treatment was not pursued due to the patient’s previous exposure and response to larotrectinib (82).

### ALK Inhibitors

The anaplastic lymphoma kinase (ALK) fusion with striatin (STRN) was identified in patient derived thyroid cancer cells in 2014, which leads to continuous MAPK signaling through MEK activation (83, 84). These mutations may be found in about 1% of ATC and 10% PDTC and occasionally in DTC (84). Although there are several ALK inhibitors that are FDA approved for lung cancer, none have been approved for thyroid cancer. There are, however, case reports of the successful use of ALK inhibitors in anaplastic thyroid cancer (85, 86).

### Iodine Re-Sensitization

As covered previously, iodine avid DTC has a much better prognosis than disease which is iodine resistant, even with distant metastasis, both due to the prognosis associated with the underlying biology of the disease and the effectiveness of RAI treatment. Consequently, researchers have tried various modalities to resensitize RAI-R TC including the administration of retinoids and anti-diabetic medications among others without consistent success (87, 88).

However, recently some targeted therapies have shown some initial promising results in promoting iodine uptake in previously refractory disease, specifically BRAF, NTRK and MEK inhibitors (51, 52, 82, 89–91). This may be explained mechanistically as described previously: Activating mutations (e.g. BRAF V600E) along the MAPK and/or PI3K pathways leads to inhibition of transcription factors important in the production of NIS. Thus, inhibiting agents may lead to an increase of NIS expression at the plasma membrane and increase the active transport of iodine into thyrocytes (14, 15). In 2013, Ho et al. evaluated 20 patients with RAI-R DTC with WBC 4 weeks after being treated with the MEK inhibitor, selumetinib. Twelve of the 20 patients showed increased iodine avidity and 8 reached the dosimetry threshold for RAI. The majority had a partial response (5) and the rest had stable disease at 3 months post treatment. All had a reduction in their thyroglobulin level (51). Similarly, Rothenberg demonstrated increased iodine avidity in 6 of 10 patients with BRAF V600E mutations treated with dabrafenib. In a retrospective study out of MD Anderson, 13 patients were treated for a longer duration (average 14.3 month) with either a BRAF or MEK inhibitor before a diagnostic WBS (89). Nine of the patients went on to RAI and all 9 patients had durable disease control at median follow up of 8.3 months (90).

Interestingly the multi-kinase inhibitors sorafenib and lenvatinib do not appear to increase iodine avidity (92). This may be explained by their predominate targets (VEGFR, PDGFR) being further upstream of the molecular control of NIS expression and additional RTKs may lead to the activation of the MAPK/PI3K pathway (for example HER signaling).

### Future Targeted Agents

Histone deacetylase (HDA) are enzymes that remove acetyl groups from lysine residues from the N terminus of DNA. Activated BRAF has been shown to promote histone deacetylation of the NIS promoter, thus decreasing expression of NIS (63, 93). There have been some promising results in *in-vivo* studies involving ATC treated with HDA inhibitors such as suberoyanilide hydroxamic acid, N-Hydroxy-7-hepanomide, butyrate among others (94). Valproic acid in part acts as an HDA inhibitor and was evaluated in a phase II clinical trial in the treatment of FTC but did not show antitumorigenic activity and did not increase iodine sensitivity (95). In addition, romidepsin is a potent and specific HDA inhibitor, but also did not show anti-tumor effects in a phase II clinical study, thought it may increase iodine sensitivity (96).

A subset of DTC will express somatostatin receptors, which have theorized as potential target for peptide receptor radionuclide therapy (PRRT). Similar to RAI, radiolabeled somatostatin analogs bind to receptors on the tumor and emit local cytotoxic effects as they degrade. This can be utilized in imaging modalities and potentially for treatment. Most available literature focuses on MTC as this is of neuroendocrine origin and has much higher expression of somatostatin receptors. ^68^Ga-DOTATOC is a somatostatin analog that is FDA approved for imaging in metastatic MTC. The largest series evaluating its imaging and therapeutic use in DTC was 41 patients with progressive iodine resistant disease who underwent imaging with ^68^Ga-DOTATOC positron emission tomography to select patients with significant somatostatin receptor expression. Eleven patients ended up being treated with PRRT (90Y-DOTATOC). Two patients had a partial response, and 5 had stabilization of disease, but the PFS was only 3.5 to 11.5 months. Adverse events included nausea, asthenia, and transient hematologic toxicity, and one patient had permanent renal toxicity (54).

Human epidermal growth factor receptors 2 and 3 (HER3) amplification in thyroid cancer has been evaluated in many series (97). This has been an active area of research as commercially available targeted therapies have proven vital in the treatment of other cancers. They are known upstream participators in both the MAPK and PI3K signaling and have been shown to participate in tumor escape from BRAF inhibitors (18). An ongoing clinical trial is assessing dabrafenib in combination with HER2/EGFR inhibitor lapatinib (NCT 01947023) in RAI-R TC with results expected in July of 2022.

### Immunotherapy

Compared to other cancers, the mutational burden on thyroid cancer is relatively low and thus they are typically poorly immunogenic (64). However, the advent of immune checkpoint inhibitors has sparked a new interest in studying the thyroid tumor microenvironment in the hopes of identifying tumors that may be more susceptible to immunotherapy. The over-expression of immune checkpoint program cell death protein 1 and ligand (PD1/PDL1) in thyroid cancer is uncommon but may be useful biomarker in predicting invasive or aggressive disease (98, 99). In a phase Ib trial, 22 patients with PD-L1 positive papillary or follicular thyroid cancer were administered pembrolizumab every 2 weeks for 24 months or until toxicity or confirmed progression. No treatment related discontinuations occurred, and most AE were grade 1 or 2. The objective response rate was 9%, with duration lasting 9-20 months (55).

PDTC and ATC are likely to have a higher mutational profile and thus may be more susceptible to immunotherapy (64). In fact, PD-L1 was shown to be expressed in 28.6% of ATC patients (100) and may even have a higher expression than many other solid tumors (101, 102). Those with a BRAF-mutated ATC may have an even higher expression. There are multiple open clinical trials evaluating PD-1/PD-L1 in inhibitors in ATC (103). Recently Capdevila et al. reported on a phase I/II study evaluating PD-1 inhibitor, spartalizumab in the treatment of ATC. Forty-two patients with ATC were enrolled, of which 28 had PD-L1 expression. The overall response rate was 19%, but was 29% in the PD-L1 positive cohort and 0% in the PD-L1 negative group. The 1 year survival in PD-L1 positive population was 52.1% (104). In another study, pembrolizumab along with chemotherapy (docetaxel/doxorubicin) was given with radiation therapy to 3 ATC patients with unresectable tumors, with satisfactory responses, but unfortunately all died within 6 months and 2 of 3 may have died from fatal pulmonary complications possibly related to treatment (56). There is retrospective evidence that pembrolizumab may be useful as a salvage for ATC patients who have progressed through TKI or BRAF/MEK inhibitors (33, 105).

Cytotoxic T lymphocyte antigen 4 (CTLA-4) is another checkpoint receptor expressed by tumor cells to suppress an anti-tumorigenic immune response. CTLA-4 inhibitors are also being investigated in clinical trials involving ATC and other aggressive thyroid cancer [NCT03246958 (103)]. Initial results of nivolumab plus ipilimumab in the treatment of aggressive thyroid cancer was published in an ASCO abstract in June 2020. In the phase II study 49 patients were treated: 32 had DTC, 10 ATC and 7 had MTC. Among the DTC patients, 3 had a partial response, and 1 had a near total response. Three patients with ATC had a partial response and 2 had no evidence of disease at 13 and 26 months (106).

## Limitations

Despite remarkable progress there continue to be 2 broad categories of limitations in both the application and understanding of novel targeted therapeutics in thyroid cancer: development of resistance and managing or limiting adverse effects.

### Response Rate and Resistance

Despite our detailed understanding of the molecular underpinning of the tumorgenesis in thyroid cancer, clinically it is often difficult to explain why one BRAF mutated RAI-R TC will respond to an inhibitor and another will not. Furthermore, one tumor may develop a drug resistance in as little as a few months and another may go years. However, there are instances when resistance has been associated with development of new mutations. For example, newly acquired secondary RAS mutations have been found in thyroid cancer patients treated with BRAF inhibitors and may act as an escape mechanism (20, 107). Research should be focused on clinical predictors of response and success for a given treatment.

Combination therapies may be crucial for a durable response. This has been shown in BRAF mutated melanoma treated with dabrafenib in combination with trametinib, though not in RAI-R TC (48, 108, 109). Though the best available data examined the combination therapy in ATC. The benefit of combining BRAF with other inhibitors (HER2/mTOR) have been hypothesized. Others have shown dabrafenib/trametinib in combination with immunotherapy may provide a more durable response (33). Additionally, TKI combinations with specific protein inhibitors and immunologics are being studied in clinical trials. As testing becomes more common and granular these results can be integrated with clinical trial data to better understand which single therapeutic or combination may be the best treatment option for varying types of RAI-R TC.

### Side Effects

Toxicities across TKIs are relatively consistent and include hypertension, fatigue, diarrhea, proteinuria, nausea and vomiting, skin reaction/rash. Hypertension appears to be the most common adverse event. In the SELECT trial, nearly 70% of patients experienced hypertension requiring treatment, and 40% had a grade 3 severity or greater. In patients with blood pressure that cannot be maintained below 140/90, or who require addition of multiple antihypertensives to pretreatment baseline, dose holding or discontinuation of TKI may be necessary (65). Palmar-plantar erythrodysthesia syndrome (PPES) is another toxicity that can occur with most TKIs, indicative of painful redness, swelling and often blisters occurring on the palms and soles. Its etiology is uncertain and has been associated with many types of cytotoxic agents. Management is largely symptomatic with dose interruptions for severe cases. Other muco-cutaneous adverse events are common including rash, alopecia, and oral stomatitis. Hyperpigmentation can be seen with sorafenib, while hypopigmentation has been reported with sunitinib and pazopanib among others. Thrombocytopenia, gastric hemorrhage, epistaxis, are also relatively common. Anti-FGFR/VEGFR targeted TKIs may interfere with wound healing, so perioperative holding of the medication is prudent. Much has been written describing dosage optimization for sorafenib and lenvatinib based on adverse events (65). Less is known about cross reactivity across TKIs, but if a patient is unable to tolerate one, another medication within this same class may be trialed. Most severe reactions occur within a short time frame after initiation. In an update to the SELECT trial it was noted that, although a grade 3 or greater AE occurred in 75.9% in the initial trial, this only increased by 5% in the subsequent 3 years of analysis. This suggests that most severe AE occur early in the course of treatment, and if managed patients may be continued on the medication for a prolonged period of time (61, 62). There is also some data that may demonstrate some of Japanese or Chinese decent may have a higher percentage of polymorphisms in specific transporters involved in the clearance of lenvatinib (110, 111) and therefore they potentially have higher blood concentrations and greater toxicity at a given dose. Other side effects may be worse in certain ethnicities, for example pazopanib associated hypopigmentation in those with dark skin tones (112).

Dabrafenib and vemurafenib have been associated with fatigue, rash and other skin reactions, hypertension (low less common than TKIs), pyrexia, diarrhea, and formation of cutaneous squamous cell cancers along with other skin growths (113). The addition of trametinib to dabrafenib may lead to more frequent fever, fatigue, diarrhea, hypertension, but decreases the rate of skin cancers (113). Dose reduction has also been suggested based on degree of side effect (113). In a clinical trial with 563 patients with metastatic melanoma treated with dabrafenib or combination dabrafenib/trametinib, 98% reported an adverse event, and 18% of patients had to discontinue treated because of a side effect. Of the 59 patients who remained in the trial and progression free for 5 years, 52 (88%) were able to tolerate dabrafenib, trametinib, or both. Among the 161 patients that were alive at 5 years, 69 continued to receive dabrafenib, trametinib, or both (114). This suggest that long term tolerance is possible for patients who respond to therapy. Some data may suggest a lower toxicity profile in dabrafenib compared to vemurafenib (115).

Selpercatinib and pralsetinib seem to have a lower toxicity profile than other FDA approved targeted therapies. Of the 531 patients treated with selpercatinib in a phase 2 clinical trial, only 21% had a grade 3 or higher adverse event and only 2% had to discontinue treatment secondary to a side effect (38). Most common AE was hypertension (at 21%), followed by alanine aminotransferase level increase, aspartate aminotransferase increase, hyponatremia, diarrhea.

Overall targeted agents appear to have less severe and long-term adverse events than conventional cytotoxic chemotherapeutic agents. However, they should be considered as chronic therapies, and therefore, AEs must be aggressively managed to prolong treatment duration in those exhibiting a clinical response. Unfortunately, all novel therapeutics have multi-system side effects and many patients will experience a significant AE within months of treatment initiation. Counselling before beginning therapy and continued monitoring is critical. Minor toxicities can be managed with proactive strategies, such as aggressive blood pressure management for TKI-associated HTN or antiemetics for nausea and vomiting. More severe and intolerable reactions must be managed with dose adjustments, medication holds, or treatment discontinuation.

## Conclusions

The available therapeutic landscape for the treatment of iodine resistant thyroid cancer is rapidly evolving. Molecular genetic profiling of biologically and clinically aggressive-behaving thyroid cancer has become standard of care. More than ever, thyroid cancer treatment can now be highly personalized based on the genetic tumor profile. The evolution from a biologic understanding of oncogenesis to the application of novel therapeutics is a model of translational science, and innovation will continue to arise from both improved understanding of thyroid-specific oncogenesis as well as application of genetic and targeted therapeutic discovery from other types of cancers. Many currently ongoing preclinical and clinical studies and trials will help further refine this treatment landscape. While there remain limitations in contemporary understanding and application of thyroid genetics and targeted therapies, the slope of the curve of progress in targeted therapeutics for radioactive iodine refractory thyroid cancer continues to rapidly increase.

## Author Contributions

TF: Manuscript writing, editing. MC: Manuscript editing. MZ: Manuscript editing. All authors contributed to the article and approved the submitted version.

## Conflict of Interest

The authors declare that the research was conducted in the absence of any commercial or financial relationships that could be construed as a potential conflict of interest.
